# Parrish’s Practical Pharmacy

**Published:** 1874-09

**Authors:** 


### Bibliographical.

---

A Treatise on Pharmacy: Designed as a text-book for the student, and as a guide for the physician and pharmacist, containing the officinal and many unofficinal formulas, and numerous examples of extemporaneous prescriptions. By Edward Parrish, late Professor of Theory and Practice of Pharmacy in the Philadelphia College of Pharmacy; member of the Academy of Natural Sciences of Philadelphia, and of the American Pharmaceutical Association. Fourth Edition, enlarged and thoroughly revised, by Thomas S. Wiegand, graduate of the Philadelphia College of Pharmacy. With two hundred and eighty Illustrations. Philadelphia: Henry C. Lea. 1874. Octavo, leather, pp. 977. Price, $6.50. Cloth, $5.50.

This work of the late Professor Parrish is the outcome of many years' experience as a teacher of pharmacy, first in his private laboratory and lecture-room, as a teacher of medical students, and afterwards as professor in the Philadelphia College of Pharmacy, in teaching students of pharmacy. It is a fitting and enduring monument of his industry, as well as of his practical good sense and intimate knowledge of the wants of American students. As a text-book, in its appropriate department, it stands singly and alone; it has no peer in the English tongue.

The scope covered by the table of contents is entirely too wide for us to attempt any analysis of it in the limits of a book notice. Nor is it needful; the student who desires a text-book upon pharmacy has at present no option. for this is the well-recognized standard in America, and is likely so to remain for years to come.

The editor, Mr. Wiegand, in the present edition, has brought the work fully up to the demands of the present day.

